# HPV: Immunology lessons from an ancient oncotarget

**DOI:** 10.18632/oncotarget.26606

**Published:** 2019-02-12

**Authors:** Sri Krishna, Karen S. Anderson

**Affiliations:** Karen S. Anderson: Biodesign Center for Personalized Diagnostics, Arizona State University, Tempe, AZ, USA

**Keywords:** HPV, immunology

Human Papillomavirus (HPV) is a sexually transmitted double-stranded DNA virus that has co-evolved with modern humans over the past 100,000 years [1]. Although there are over 100 different HPV subtypes [1], only a subset are oncogenic causing cervical cancer and cancers of other histologies [2]. Of these, HPV16 is implicated in >60% of cervical cancers, and in >80% of oropharyngeal cancers [2]. While HPV-induced cervical cancer remains a global threat, there is a rising incidence of HPV-induced head and neck squamous cell carcinomas (HNSCCs), rapidly overtaking both smoking-induced HNSCCs as well as cervical cancer cases in the US [2].

There are a variety of factors that make HPV16 an ideal target for cancer immunotherapy: 1) HPV-gene expression plays a crucial part in the viral-driven malignancy, and is therefore likely to be retained as an early driver event in every cancer cell [3]. 2) The immune system can eliminate HPV-lesions, since not all HPV16-seropositive individuals develop cancers. 3) Prophylactic vaccines against HPV-infections are very effective; however, the vaccine uptake has been low in adolescents (49%), however vaccine uptake has been low in adolescents, and the full potential of HPV vaccination will not be felt for decades.

The long lag time from HPV infection to the development of cancer, readily detected by serology [4], provides ample opportunity to boost pre-existing HPV-specific T-cell (CTL) immunity to enhance viral clearance. HPV-specific CTLs have been described primarily in cervical cancers, but less well in context of HPV+HNSCCs [5, 6]. T-cell vaccines and adoptive cell therapies targeting the HPV16 E6 and E7 genes have been previously reported to have mixed success in precancerous stages of cervical cancer [5]. In comparison, HPV+HNSCCs have no known precancerous lesions, but have a higher expression of the HPV E2 and E4 genes compared to cervical cancers, with lower rates of HPV-integration into the human genome [3, 7]. Thus, the immunobiology of HPV+HNSCCs may differ from cervical cancers.

In a recent study, we addressed the immunogenicity of HPV16- E2, E6, E7 antigens, and studied the mechanisms of HPV16 immune evasion in HPV+HNSCC patients [7]. We found that HPV16-E2 specific T-cell immunity was abundant in the peripheral blood of HPV+HNSCC patients, and perhaps surprisingly higher, relative to E6 and E7 T-cell immunity which are the traditional targets of HPV T-cell therapies in cervical cancer. We identified 16 strong and 29 moderately immunogenic HPV16 CTL-epitopes across 12 common HLA class I supertypes. Knowledge of these HPV16-epitopes, their HLA-restrictions, and immunogenic domains of the HPV-antigens can aid the design and development of HPV-specific immunotherapies.

**Figure 1:**
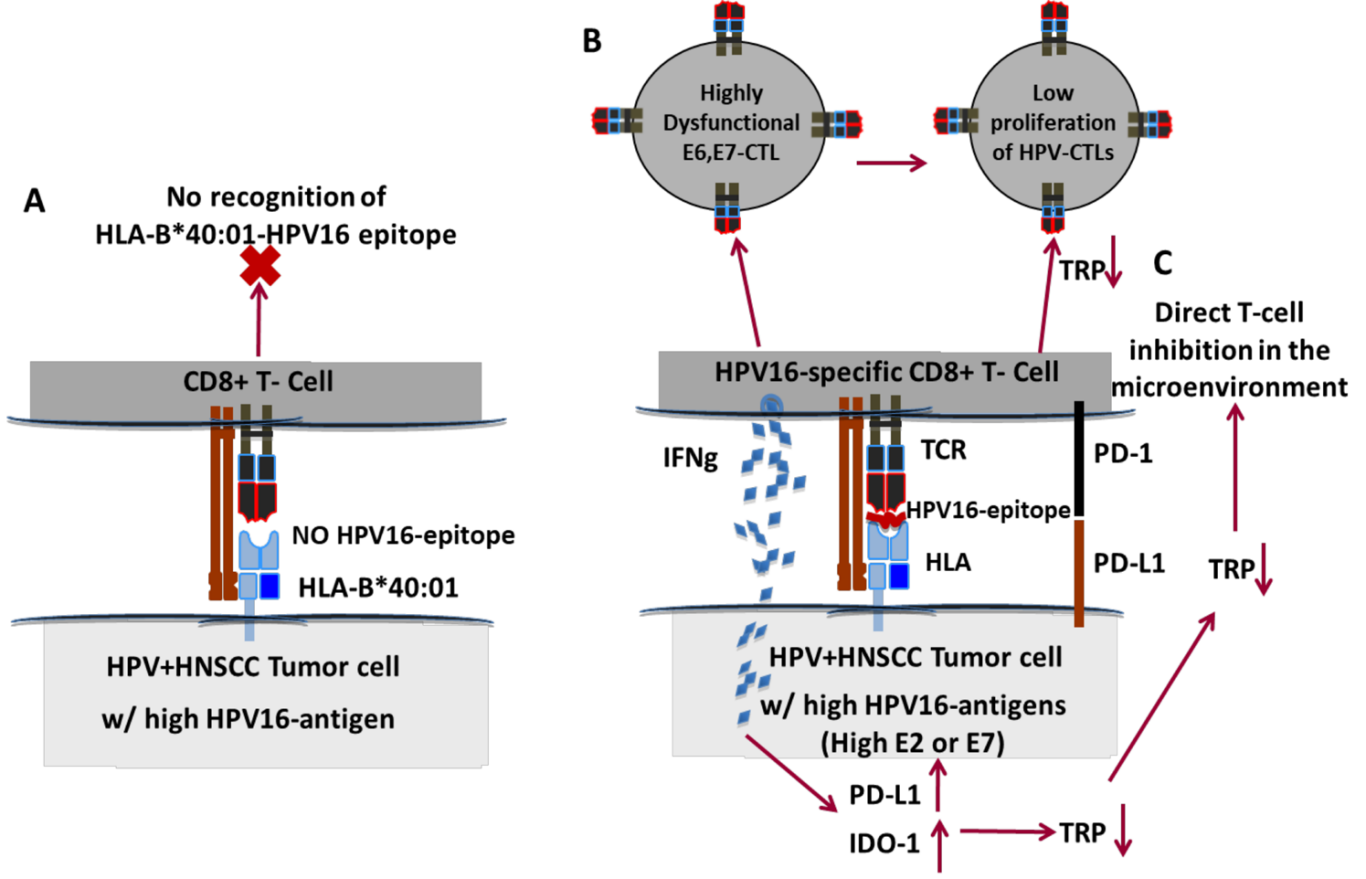
Proposed mechanisms of HPV16 immune evasion in HPV+HNSCCs **A.** Lack of tumor cell recognition because of absent HPV16-epitope. **B.** Dysfunction of E6, E7 CTLs in the tumor microenvironment leading to outgrowth of clones expressing E6/E7. **C.** E2, E6, E7 CTLs are able to infiltrate the tumor in response to high antigen expression but are directly inhibited by PD-1/PD-L axis and IDO-1 expression leading to impaired tumor targeting by HPV-CTLs. IFNg - Interferon gamma. TRP- Tryptophan. TCR - T-cell Receptor.

A long history of co-evolution of alphapapillomaviruses with humans means that HPV may possess multiple mechanisms of immune evasion, and this was confirmed by our study [7]. We found that certain HLA-alleles, such as HLA-B*40:01 and HLA-B*07:02 had very few predicted HPV16 binders and thus patients with these HLAs were at an increased risk of being represented in the HPV+HNSCC cohort relative to HPV- HNSCC cohort (Figure 1). We further found that HPV16 E6 and E7-specific CTLs in the periphery had high levels of T-cell inhibitory markers such as CD39 and PD1, consistent with the impaired E6- and E7-specific T-cell function observed by us and others in patients. We also explored mechanisms of immune evasion in HPV+ HNSCC from the tumor side. We found that HPV16 antigen expression highly correlated with tumor intrinsic dysfunctional gene expression, including up-regulation of immune regulatory PD-L1 and IDO1 in the tumor cell. These studies were validated in both primary tumor samples using transcriptome data, and in *in vitro* model systems. We exploited these findings to then demonstrate that blocking PD-L1 and IDO1 pathways can enhance HPV-specific T-cell targeting of HPV+ HNSCCs *in vitro*.

Based on these studies, one can envision at least three mechanisms of HPV-specific immune dysfunction observed in HPV16+ HNSCCs. First, the lack of appropriate HLA-HPV-epitope presentation can lead to failure of tumor recognition by T-cells (Figure 1A). Second, E6 and E7 CTLs are highly dysfunctional in HPV+ HNSCC patients, leading to tumor immune escape (Figure 1B). Third, even if HPV-CTLs infiltrate the tumor in response to high HPV-antigen expression in the microenvironment, tumors upregulate PD-L1 and IDO-1 to directly inhibit HPV-specific CTL proliferation and function (Figure 1C).

Future studies can leverage our findings to improved targeted HPV-immunotherapy for HPV+ HNSCCs. For instance, in addition to E6 and E7, E2-specific CTLs need to be pursued in context of HPV-therapeutic vaccines or adoptive T-cell therapies [5-7]. We predict that patients with tumors harboring episomal HPV16 genomes, with high expression of E2, will have impaired response to E6/E7 T-cell therapies, since E2 can transcriptionally repress the expression of E6/E7 [3]. In contrast, highly integrated HPV tumors may have limited, or even pathologic, responses to E2-targeted therapies as E6 and E7 are de-repressed. We also propose that the dynamics of E2, E6 and E7 expression will lead to significant tumor (and immune) heterogeneity in HPV+ HNSCCs warranting further studies. Lastly, the combination of PD-1 blockade with IDO-1 to expand HPV-specific CTLs ex vivo should be further explored. We anticipate that there is much to learn about the immunobiology of this ancient virus which could inform us about cancer immunotherapy.
